# Office Visitor

**Published:** 1906-03

**Authors:** 


### OFFICE VISITOR.

It was about closing up time after a day's work; sitting at our desk looking over the record of the day's work and trying to hurry up to write an important delayed letter, our equanimity was disturbed by the ring of the door bell. The office girl having departed, we ejaculated a very pious word and answered ourself. A thin, weazened faced man greeted us, and in a rapid manner asked if he could have a few minutes of our time to present something very important to us in our practice of dentistry. Hardly waiting an assent he walked in rapidly, took a seat rapidly, and rapidly began his lecture, for such was the nature of his harangue before the business feature of his mission was presented. He had a father and that father was the originator of this wonderful method under description. His dad was doing the next state, while the son was doing ours and trying to do us, we found out.

"The physicians believe in preparing patients for operations, consequently dose them for days before subjecting them to the keen cut of the knife, for though an anaesthetic is used, unless the physical condition is in proper condition, recovery is not so rapid, and danger of shock is great. Athletes go into training before venturing to withstand the severe strain and punishment of a fight, a test of strength or the contests of sprinting or jumping, etc. By a series of laborious and tedious experimentation, that dad of his had perfected a system of dosing "whereby a dental patient is rendered immune from the pangs of operations on teeth. A tablet or two of a medicament of his so many times a day for several days previous to the appointment would enable patient to submit to excavating, burring and grinding his teeth with a smile." Then was produced a volume of signatures of many of the most prominent dentists of the land and of neighboring cities and towns, as purchasers of the medicine. After the rapid exposition of the wonderful rapid remedy of the twentieth century and rapid exhortation to invest seven dollars in its benefits, we got a slight chance to put in a word edgewise. A question or two rattled him somewhat but with resource and rapidity he replied to our inquiries semi-satisfactorily, "Oh, well, Doctor, it is too good a thing to cast aside for the small sum of seven dollars, but to allay your doubts, pay me half the money down and I will send you the tablets with full instructions and when you are satisfied on trial that it is no fake you can send me the balance due." Then we turned our batteries of argument on him with a rapid fire along the whole line and wound up by the climax that being an editor of a dental journel had kept abreast of society proceedings and magazine articles and had seen no reference whatever of his most wonderful scheme in either book or story. Whereupon he referred us to an article on therapeutics from the pen of an eminent professional dentist of a very rapid city of the Northwest. We assured him we had read the article and had clipped from it; but challenged him to point to one point in it in reference to his process of medication, whereupon he packed his numerous letters of recommendation and rapidly retired from the office to catch the next train. Since his departure we learn the beguiled in our midst are still awaiting the arrival of the tablets, all gotten on half pay.

